# Assessment of Soft Computing Techniques for the Prediction of Compressive Strength of Bacterial Concrete

**DOI:** 10.3390/ma15020489

**Published:** 2022-01-10

**Authors:** Fadi Almohammed, Parveen Sihag, Saad Sh. Sammen, Krzysztof Adam Ostrowski, Karan Singh, C. Venkata Siva Rama Prasad, Paulina Zajdel

**Affiliations:** 1 Civil Engineering Department, Shoolini University, Solan 173229, India; 2 Civil Engineering Department, Chandigarh University, Mohali 140413, India; parveen12sihag@gmail.com; 3 Department of Civil Engineering, College of Engineering, University of Diyala, Baquba 32001, Iraq; saad1234engineer@gmail.com; 4 Faculty of Civil Engineering, Cracow University of Technology, 31-155 Cracow, Poland; krzysztof.ostrowski.1@pk.edu.pl (K.A.O.); paulina.zajdel1@pk.edu.pl (P.Z.); 5 Civil Engineering Department, National Institute of Technology, Hamirpur 177005, India; karans72@gmail.com; 6 Civil Engineering Department, St. Peters Engineering College, Dhulapally, Maisammaguda, Medchal, Hyderabad 500100, India; cvsrprasad90@gmail.com

**Keywords:** bacterial concrete, compressive strength, soft computing techniques, support vector regression, M5P, random forest, Random Tree, artificial intelligence

## Abstract

In this investigation, the potential of M5P, Random Tree (RT), Reduced Error Pruning Tree (REP Tree), Random Forest (RF), and Support Vector Regression (SVR) techniques have been evaluated and compared with the multiple linear regression-based model (MLR) to be used for prediction of the compressive strength of bacterial concrete. For this purpose, 128 experimental observations have been collected. The total data set has been divided into two segments such as training (87 observations) and testing (41 observations). The process of data set separation was arbitrary. Cement, Aggregate, Sand, Water to Cement Ratio, Curing time, Percentage of Bacteria, and type of sand were the input variables, whereas the compressive strength of bacterial concrete has been considered as the final target. Seven performance evaluation indices such as Correlation Coefficient (CC), Coefficient of determination (R^2^), Mean Absolute Error (MAE), Root Mean Square Error (RMSE), Bias, Nash-Sutcliffe Efficiency (NSE), and Scatter Index (SI) have been used to evaluate the performance of the developed models. Outcomes of performance evaluation indices recommend that the Polynomial kernel function based SVR model works better than other developed models with CC values as 0.9919, 0.9901, R^2^ values as 0.9839, 0.9803, NSE values as 0.9832, 0.9800, and lower values of RMSE are 1.5680, 1.9384, MAE is 0.7854, 1.5155, Bias are 0.2353, 0.1350 and SI are 0.0347, 0.0414 for training and testing stages, respectively. The sensitivity investigation shows that the curing time (T) is the vital input variable affecting the prediction of the compressive strength of bacterial concrete, using this data set.

## 1. Introduction

Concrete is located in the second place (after water) as the most consumed material. Like most construction materials, it is prone to microcracking and has air voids in the structure. Microcracks and micropores in concrete are very undesirable because they provide an open path for the ingress of water and other harmful substances. They can also cause corrosion of steel bars and deterioration of concrete.

Rapid development of buildings, especially in developing countries causes high energy consumption, environmental pollution and exploitation of resources. These behaviors directly affect the comfort and health of building residents [1,2]. Considering the construction development, concrete strength, concrete durability, and eco-friendliness with industrial materials (fly ash, blast furnace slag, metakaolin, silica fume, etc.), continuous research in the field of concrete technology has led to the growth of unique concrete.

High-strength concrete (HSC) is a type of concrete containing special additives in mixtures [3,4] and it represents a crucial step in the development of concrete technology [5]. The durability also plays an important role in concrete structures based on HSC. Since new building materials are not needed, its durable structure with a long service life helps to save resources. It also reduces the generation of construction waste, thereby reducing the environmental pollution. According to the economic view, it decreases repair and maintenance costs [6,7,8].

Usually occurring defects are cracks caused by the following reactions, action, shrinkage, freeze-thaw, the low tensile strength of concrete, hardening of concrete, etc. [9].

This bacterial self-healing method is superior to other methods because it is biologically based, environment-friendly, cost-effective and sustainable. It has been found that urease-positive bacteria can affect the precipitation of calcium carbonate (calcite) by producing urease. The enzyme catalyzes the hydrolysis of urea into CO_2_ and ammonia, leading to pH precipitation and an increase in calcite in the bacterial environment. To avoid leaching in the channel, this innovative environmental protection technology had been first used to repair cracks. It has been found that the precipitation of calcite induced by Bacillus Pasteur and Bacillus sphaericus can effectively repair concrete cracks and improve the compressive strength [10,11,12].

The durability of concrete samples in alkaline, sulfate, and freeze-thaw environments treated by Bacillus Pasteur is improved, comparing with untreated conventional concrete samples.

The durability of concrete samples is of particular importance for aqueous environments that determine the performance of concrete structures. As reflected in the study [13,14], Ghassemi et al. conducted a study of the long-term fatigue performance of PC (polymer concrete), EPC (epoxy polymer concrete) and OCC (ordinary cement concrete) in various environments. The samples were exposed to four different environments for 6 and 12 months. It was the impact of sea water and fresh water as well as of acidic and alkaline solutions. The research has shown that the aqueous environment has a destructive effect on the properties of PC and EPC, significantly reducing fatigue life and fatigue strength compared to other environments. On the other hand, the solutions of fresh and sea water improved the fatigue strength of OCC samples after six months of exposure. In the case of sea water, the improvement of properties lasted only up to the first 6 months. The alkaline and acid solutions caused a deterioration of the fatigue resistance of OCC samples to a greater extent than of PC and EPC samples. Scientists have shown the destructive effect of the aqueous environment, especially on modified concretes. It is an important aspect for a future analysis, in the case of the bacterial concretes considered in this paper.

As per the literature reviewed, materials viz. bentonite and magnesium oxide could aid in attaining high sealing proficiency for the cracks having initial width of around 0.18 mm and act as effective sealing for cracks [15]. As reflected from the study [16], Huang et al. characterized self-healing behaviors of microcracks. C-S-H and calcium carbonate [17] have been utilized as two products for self-healing of the concrete cracks as a formation of water as well as carbon monoxide dihydrate. Utilization of organic compounds, bacteria as well as volcanic ash-coated materials proves to be another effective technique for resolving cracks issue. This method includes differentiation of biological (e.g., bacteria) factors from the chemical factors (e.g., calcium lactate) and conjoining them yields in sophisticated outcomes.

Bacteria can be used to manufacture CaCO_3_ in Biological Concrete and Self-Repair or Microbial-Induced Calcite Precipitation (MICP). It fills in any gaps that occur in the concrete. Bacillus pseudo bacillus, Bacillus sphaericus, Bacillus pasteurella, Escherichia coli, Bacillus balodurans, Bacillus sphaericus, Bacillus subtilis, Bacillus halodurans, and other bacteria can be used in concrete. These bacteria can live in an alkali-rich environment, by using the metabolic process including sulfate reduction, urea hydrolysis, and photosynthesis.

The technology can be a method of bio-mineralization used on the surface or inside of concrete. The internal method involves depositing calcite (calcium carbonate) into concrete at a specific concentration. One of the bio-mineralization processes is the Microbial-Induced Calcite Precipitation (MICP). Microbial urea hydrolyzes urea to create ammonia and carbon dioxide, which is the most important concept in this process. The pH would then rise as a result of releasing the ammonia into the atmosphere. The released carbon dioxide reacts with calcium carbonate, which accumulates in the concrete pores [18]. Kalra et al. [19] and Irigaray et al. [20] show that the relationship between concrete strength is a highly nonlinear function. Therefore, a comprehensive mathematical model is used to establish a nonlinear analysis function, which can better understand the performance of concrete, compared to the concrete composition of conventional concrete without cementing materials. Therefore, it is necessary to use its mixture composition and proportion to explore high performance computing (HPC) model.

Regression approaches and machine learning techniques have been used in recent studies to measure and forecast Compressive Concrete Strength (CCS) [21,22]. Researchers may now use modeling techniques to further infer realistic solutions, due to the growing use of modeling techniques in the engineering field [23,24,25].

In CSS estimation problems, linear and nonlinear regression (LR and NLR) approaches are commonly used. In 2002, Bhanja and Sengupta [22] conducted a report on CCS, estimating the strength of 28-day concrete using a 5 percent to 30% silica fume substitute as a cementitious material. The role of concrete dimensionless variables in CCS prediction has been expressed in the success of their nonlinear model. In a subsequent study [26], the role of multilinear regression in predicting 28-day CCS was emphasized. To measure the CCS of construction sites more rapidly and reliably, many cementing materials must be analysed.

NLR can solve a wider range of classification. Regression trees and regression problems (CART) have been commonly used to predict the relationship between variables, according to their graphical representation and simplicity [21,27]. In CART, the same predictor variable could be used at different levels, and compared to other modeling techniques, what is considered multiple times in classification trees and regression trees (RT).

Artificial neural networks (ANN) have been used in a variety of studies to model the relationship between concrete properties and their components [21,28,29], to predict CCS, Water, cement, super plasticizer, silica fume, coarse aggregate, and fine aggregate composition, as well as 25 different artificial neural network architectures. In 28 days without slump CCS, Sobhani et al. compared the prediction results of NLR, ANN, and the inference method model based on the adaptive network [30]. They tested multiple structures of each model and found that the adaptive network-based reasoning method and ANN produced acceptable CCS prediction results, when compared to the NLR-based model.

In 2007 Gupta took the lead in using support vector machines (SVM) to forecast CCS, owing to the widespread popularity of machine learning techniques for basic output prediction [31]. The potential of SVM as an efficient technique for modeling CCS using a limited number of samples is demonstrated in this analysis. The research was also conducted by Yan and Shi to demonstrate the effectiveness of SVM in predicting concrete results [32]. According to their findings, SVM performed well, in comparison to other models. However, the experimental data used in the analysis, like most others, seldom had a minimal compressive strength boundary.

Support vector regression, M5P, Random Forest (RF), Random Tree (RT), and Reduced Error Pruning Tree (REP Tree) techniques have all enhanced their efficiency in various fields. The capacity of these soft computing techniques for the estimation of the compressive strength for bacterial concrete is evaluated in this analysis and compared to a multiple linear regression-based equation. According to the authors’ best knowledge, the efficiency of these techniques for the prediction of the compressive strength for bacterial concrete has not been compared yet. This manuscript employs five statistical indices and one graphical tool (the Taylor diagram) to evaluate the performance of developed models.

## 2. Research Significance

Regression approaches and advanced machine learning techniques allow the realistic prediction of concrete properties. The present research permits to verify which of the tested computational models enables the best prediction of the compressive concrete strength (CCS) for bacterial concrete. Correct prediction of concrete properties will enable the efficient and economical design of durable structures, minimizing the time of selecting the appropriate material, as well as the time and resources committed for repairs. The proposed forecasting techniques also allow civil engineering scholars of the smart design new materials.

## 3. Review of Regression and Soft Computing Techniques

### 3.1. Multiple Linear Regression

Using observed data, multiple linear regression is utilized to discover the link between input and output variables. For nonlinear and difficult issues, MLR is frequently used [33,34]. MLRs are a multivariate statistical approach that fits a linear equation to observed data to represent the linear correlations between the dependent variable y and two or more independent variables. Each response of independent variables is related with the value of the dependent variable y. The equation for y’s regression can be written as follows:

(1) $$y=m_{1}x_{1}+m_{2}x_{2}+m_{3}x_{3}+\cdots +m_{n}x_{n}+C$$

where *y* is the dependent variable, $x_{1}$, $x_{2}$…$x_{n}$ are the independent variables, $m_{1}$, $m_{2}$…$m_{n}$ are the regression coefficients, and C is constant.

MLR models were the usual method for estimating responses between a dependent variable and various independent factors where the dependent variable and independent variables had a linear connection [35].

### 3.2. Random Forest

Breiman was the first to suggest the random forest algorithm (1996) [36]. It is a highly adaptable algorithm that is used successfully to a variety of engineering problems.

Random Forest (RF) is a tree-structured classifier that consists of a group of classifiers. Bagging and random feature selections are two important machine learning techniques features used by RF.

The target class is the target pattern of each tree as the target class in this machine learning classifier, which includes several decision trees.

RF is a step away from bagging. When growing a tree, RF will randomly pick a subset of features to break at each node rather than using all of them. RF uses Out-of-Bag (OOB) samples to perform cross-validation in parallel with the training phase in order to test the random forest algorithm’s prediction accuracy.

Depending to (Breiman 2001) [37] the random forest algorithm is easy to use, has a low learning curve, and has higher prediction accuracy. Two user-defined parameters must be tuned for the best solution. The number of trees increases and the number of input parameters are user-defined parameters. For model development, the trial-and-error method is used.

### 3.3. M5P Model

M5P is a straightforward algorithm for predicting complex and nonlinear problems. Quinlan (1992) [38] introduced the M5 tree as a new tree algorithm for predicting complex problems with several data sets and input variables. The algorithm also includes pruning to reduce the chance of overfitting. Instead, each node is divided to gain more information, and the variance of the class value in the subset up to each branch is smaller. A division norm is used to create the basic tree model, which provides the standard deviation of the class value extended to the node. At each node, a linear relationship is formed using this approach. The algorithm created a good tree structure with high prediction accuracy [39,40,41,42,43,44]. It has been proven many times that computational procedures based on uncertainty modelling and probabilistic structural analysis are used in the analysis of engineering structures [45].

By classifying or splitting the entire data space into many subspaces, the tree algorithm allocates a linear regression function at the terminal node and fits a multiple linear regression model to each subspace. The M5 tree method can handle very high dimensionality and deals with continuous problems rather than discrete ones. It shows the piecewise details of each linear model that was built to approximate the data set’s nonlinear relationship. Information about the partition norm of the M5 model tree has been obtained based on the error calculations of each node. Calculate the standard deviation of the class value that reaches the node to determine the error. To break at this node, choose the attribute that maximizes the reduction of expected errors caused by the test of each attribute. The following formula is used to measure the standard deviation reduction (*SDR*):

(2) $$SDR=sd\left(K\right)-\sum \frac{\left|K_{i}\right|}{\left|K\right|}sd\left(K_{i}\right)$$

where,

*K*—set of instances that attain the node.
*K_i_*—the subset of illustrations that have the product of the possible set.
and sd —the standard deviation

### 3.4. Random Tree Model

The multi-strategy feature of RT allows users to obtain a very diverse set of regression models. Nonetheless, these models are tree-based, which means that training cases would be divided by tree-based models or constructed for all training cases.

For tree-based models, RT has the following main features: learning trees to minimize the square error (least squares (LS) regression tree), learning trees to minimize the absolute deviation (least absolute deviation (LAD) regression tree), selecting the probability between different models to be used in the leaves during the prediction task is pruned based on the sequence-based regression tree. RT generated a set of alternate tree models and selected one of them based on some criteria. Different methods have been used to estimate the best pruning tree from a series of alternate trees.

### 3.5. Reduced Error Pruning Tree (REP Tree)

Rep Tree generates several trees in various iterations using regression tree logic. After that, it selects the best tree from among all the trees that have been created. A representative is someone who represents another individual. When pruning a tree, the mean square error of the tree’s prediction is used as a metric. The (“REPT”) is a quick decision tree learning method that constructs a decision tree using knowledge gain or reduced variance.

### 3.6. Support Vector Regression (SVR)

The support vector machine SVM is an advanced machine learning technique for data classification that is based on statistical theory. To differentiate the two forms of samples, SVM looks for the best hyper plane. It maps the original data to higher-dimensional feature space and finds the best separation hyperplane there using a kernel function.

Support vector regression (SVR) uses sparse, kernels, and VC control of the number and number of support vectors, much like classification. SVR is proved to be an operative tool for real-valued function estimation, despite not being as common as SVM.

The (SVM) is based on statistical learning or the Vapnik-Chervonenkis (VC) theory, and it is capable of locating previously unseen data.

Kernels, sparse solutions, and VC regulation of the number and edges of support vectors are all features of support vector regression (SVR). SVR is proved to be an efficient tool for real-valued function estimation, despite not being as common as SVM. SVR is a supervised learning system that employs asymmetric loss function for instruction that penalizes both high and low miscalculations. A flexible tube with a minimum radius is shaped symmetrically around the estimation function using Vapnik’s insensitive process so that absolute values of errors less than a certain threshold are ignored above and below the estimation value. Points outside the tube were penalized in this way, but points inside the tube were not penalized. One of the key benefits of SVR is that its computational complexity is independent of the input space’s dimensionality. It also has a high prediction accuracy and excellent generalization ability.

### 3.7. Performance Evaluation Indices

Correlation coefficient (CC):

The correlation coefficient measures the intensity of a linear relationship between two variables. The correlation coefficient is always having a range between −1 and 1 in value, where 1 or −1 means complete correlation (in this case, all points are on a straight line). A positive correlation means the positive correlation between variables (the increase in one variable corresponds to the increase in another variable), while negative correlation means the negative correlation between variables (the increase in value is that one variable resembles the decrease in another variable). A correlation value comes close to 0 means that there is no correlation among variables.

Correlation coefficient:

(3) $$CC=\frac{\sum_{i=1}^{N}\left(O_{i}-\bar{O}\right)\left(P_{i}-\bar{P}\right)}{\sqrt{\sum_{i=1}^{N}{\left(O_{i}-\bar{O}\right)}^{2}}\sqrt{\sum_{i=1}^{N}{\left(P_{i}-\bar{P}\right)}^{2}}}$$

Coefficient of determination (*R*^2^):

(4) $$R^{2}={\left[\frac{\sum_{i=1}^{N}\left(O_{i}-\bar{O}\right)\left(P_{i}-\bar{P}\right)}{\sqrt{\sum_{i=1}^{N}{\left(O_{i}-\bar{O}\right)}^{2}}\sqrt{\sum_{i=1}^{N}{\left(P_{i}-\bar{P}\right)}^{2}}}\right]}^{2}$$

Root mean square error (*RMSE*):

The root mean square deviation (RMSD) or root mean square error (*RMSE*) is a widely used measure of the difference between a model’s or estimator’s expected value and the observed value (sample or population value). The root mean squared deviation (RMSD) is the square root of the root mean squared average.

Root mean square error:

(5) $$RMSE=\sqrt{\frac{1}{N}\sum_{i=1}^{N}{\left(P_{i}-O_{i}\right)}^{2}}$$

Mean absolute error:

The mean absolute error (*MAE*) is a statistic that measures the difference in error between pairs of observations that describe the same phenomenon. The calculation formula of *MAE* is represented by the Equation (6);

Mean absolute error:

(6) $$MAE=\frac{1}{N}\sum_{i=1}^{N}\left|P_{i}-O_{i}\right|$$

Nash Sutcliffe model efficiency:

The formula for calculating the efficiency of Nash-Sutcliffe is: divide the ratio of the observed time series variance by the error variance of the modeled time series. The Nash-Sutcliffe efficiency is equal to 1 (*NSE* = 1) in the case of an ideal model with an expected error variance equal to zero.

Nash-Sutcliffe efficiency

(7) $$NSE=1-\left[\frac{\sum_{i=1}^{N}{\left(O_{i}-P_{i}\right)}^{2}}{\sum_{i=1}^{N}{\left(\bar{O}-\bar{P}\right)}^{2}}\right]\;0\leq NSE\leq 1$$

O= values of the actual observations in a sample
$\bar{O}=$ mean of the values of the actual observations
P= values of the predicted observations in a sample
$\bar{P}=$ mean of the values of the predicted observations

## 4. Materials and Methodology

Materials

The materials have been used in this experimental for define the effect of bacteria on the mechanical properties of concrete. In this study, Bascillus subtilis bacteria with calcite lactate were used in different percentage such as 5%, 10%, and 15% of cement weight for M20 and M40 grade concrete.

Ordinary Portland cement of 53 grade was used and tested for various properties as per IS: 4031-1996 [46] and the physical properties shown in Table 1. Local available river sand and crushed stone sand were used as fine aggregates. Crushed granite broken stone of 20 mm nominal size was used as coarse aggregate. Properties of coarse aggregate were shown in Table 2. Fresh water was used in the manufacturing of concrete. The mixing and curing of concrete was done as per IS: 456-2000 [47].

In this research Bacillus subtilis microscopic organisms utilized which are refined at DVS Bio life Pvt Ltd. Laboratory, Hyderabad, India. Calcium lactate utilized for this examination alongside Bacillus subtilis microbes and nutrient broth. It is accessible in powder shape having white shading. The nutrient broth was set up by including 2.5 g of peptone, 2.5 g of NaCl, 1.5 g beef extract concentrate to 500 mL refined water in a cone shaped flask. This flask was secured with cotton plug and encased with silver thwart. From that point forward, the arrangement was untainted in autoclave over 20 min at 121 °C temperature. After this disinfection, the arrangement was in orange shading and polluting influence free. From that point forward, the cup opened in laminar wind current chamber and the bacillus subtilis microorganisms added to this arrangement. From that point onward, the arrangement hatched in orbital shaker at a speed of 125 rpm at 37 °C. Following one day, this arrangement was changed into whitish yellow hue. Calcium lactate (C_6_H_10_CaO_6_) were used for this work along with bacillus subtilis bacteria as nutrient broth. This material has the form of powder having white colour.

### 4.1. Design Proportions

The mix proportions for M40 and M20 grades concrete are designed using IS: 10262-2009 [48]. Materials required per one cubic meter of concrete are shown in Table 3 and Table 4.

### 4.2. Data Set

Data set: overall 128 observations have been collected from laboratory experiments. Out of 128, randomly separated 87 observations were chosen as training data set, and the rest 41 observations were taken for model validation and testing. Total data set consider 7 input variables namely Cement (C) in kg, Sand (S) in kg, Aggregate (A) in kg, Water to Cement Ratio (W/C), Curing Period (T) in days, and compressive strength of bacterial concrete is considered as output. Features of the overall data set, training, and testing data sets are listed in Table 5. Table 5 indicates that the range of cement is 340–390 kg, sand 642–736 kg, aggregate 1214–1261 kg, water-cement ratio 0.42–0.48, and curing period 7–365 days. There are two types of sand: natural sand (1) and crushed aggregate sand (2).

## 5. Results and Discussion

### 5.1. Results of Multiple Linear Regression

A Linear regression-based model is used to develop a linear relationship among independent variables and dependent variables. In this study XLSTAT software (Addinsoft, Paris, France) is used to develop the MLR model. It relies on the least square technique. The LR model is developed using training data set and its final equation is as follow:

(8) CS=−95.25+0.35 C+0.056 T+10.32 BC+3.21 K

**Figure 1:**
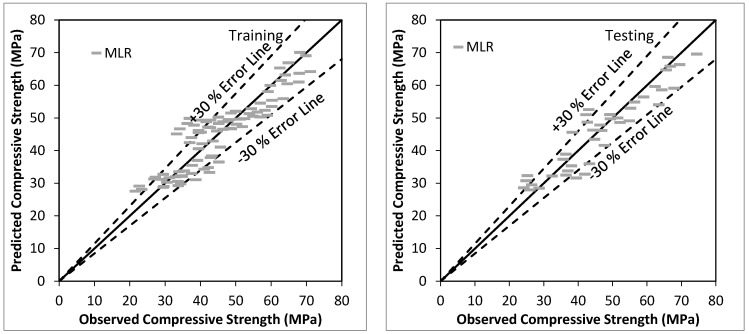
Observed vs. Predicted values using MLR based model using training and testing stage.

Figure 1 shows the agreement plot among observed and predicted values, using MLR based model for the training and testing stage respectively. The predicted values are among ±30% Error band. Overall performance of MLR based model is satisfactory for the prediction of the compressive strength of bacterial concrete with CC (0.9373), R^2^ (0.8786), RMSE (4.8727), MAE (3.9642), Bias (−0.7300), SI (0.1041), and NSE (0.8735) using testing data.

### 5.2. Results of the Tree and Forest-Based Models

**Figure 2:**
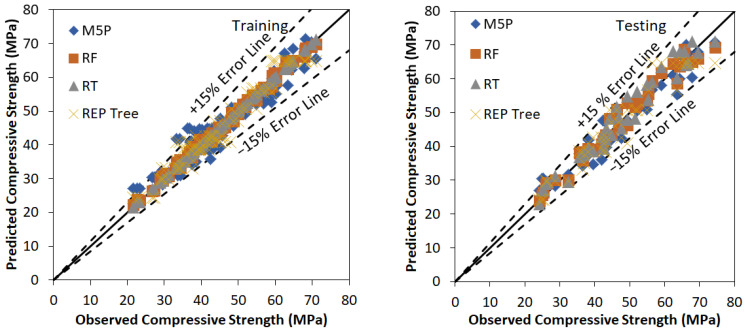
Observed vs. Predicted values using M5P, RF, RT and REP tree based model using training and testing stage.

In this investigation M5P, RF, RT, and REP based models have been developed using WEKA software to predict the values of the compressive strength of bacterial concrete. The model development is a trial-and-error process. Many trials have been carried out to find the optimum value of user-defined parameters of various tree and forest-based models. Figure 2 represents the agreement plot between observed and predicted values using RF, M5P, RT, and REP Tree based models for the training and testing stage respectively. Most of the predicted values using M5P, RF, RT, and REP tree-based models are within ±15% error band. Performance assessment indices values for various tree and forest-based models using training and testing data set are listed in Table 6. Outcomes of Table 6 conclude that the RF based model is performing better than M5P, RT and REP based models for training and testing stages with higher values of CC (0.9973, 0.9868), R^2^ (0.9947, 0.9739), NSE (0.9944, 0.9722) and lower values of RMSE (0.9010, 2.2853), MAE (0.7387, 1.8161), Bias (0.0890, 0.2327) and SI (0.0199, 0.0488) respectively. Other major outcome from this analysis is the fact that RT based model is found to be performing better than M5P and REP Tree based models with values of CC (0.9999, 0.9808), R^2^ (0.9999, 0.9620), NSE (0.9999, 0.9576) and lower values of RMSE (0.1316, 2.8224), MAE (0.0589, 2.4918), Bias (0.0000, 0.8627) and SI (0.0029, 0.0603) respectively. It has been shown that there is a difference among observed and predicted values using M5P, RF, RT, and REP Tree based models. All the tree and forest based applied models are useful to predict the values of the compressive strength of bacterial concrete.

### 5.3. Results of SVR Based Models

**Figure 3:**
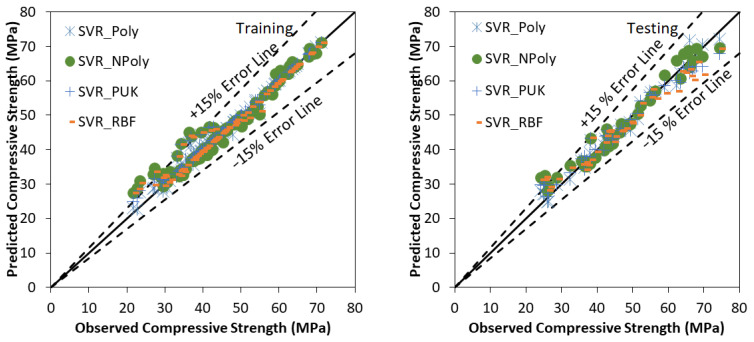
Observed vs. Predicted values using SVR based models using training and testing stage.

SVR based model development is similar to a tree or forest-based model development. Many trials have been carried out to find the optimum value of users defined parameters of various tree and forest-based models. Figure 3 presents the agreement plot among observed and predicted values using SVR based models for the training and testing stage respectively. Most of the predicted values using SVR based models are within ±15% Error band. Performance assessment indices values for various tree and forest-based models using training and testing data set are listed in Table 6. Outcomes of Table 6 conclude that the polynomial kernel function based support vector regression based model is performing better than normalized Poly kernel, Pearson VII, and radial basis kernel function based models with CC (0.9919, 0.9901), R^2^ (0.9839, 0.9803) NSE (0.9832, 0.9800) and lower values of RMSE (1.5680, 1.9384), MAE (0.7854, 1.5155), Bias (0.2353, 0.1350) and SI (0.0347, 0.0414) for training and testing stages, respectively. In Figure 3 SVR_Poly denotes the Polynomial kernel function based support vector Regression based model, SVR_NPoly denotes the Normalized Poly kernel function based support vector Regression based model, SVR_PUK denotes the Pearson VII kernel function based support vector Regression based model and SVR_RBF denotes the Radial basis kernel function based support vector Regression based model.

### 5.4. Comparison among Regression and Soft Computing-Based Models

**Figure 4:**
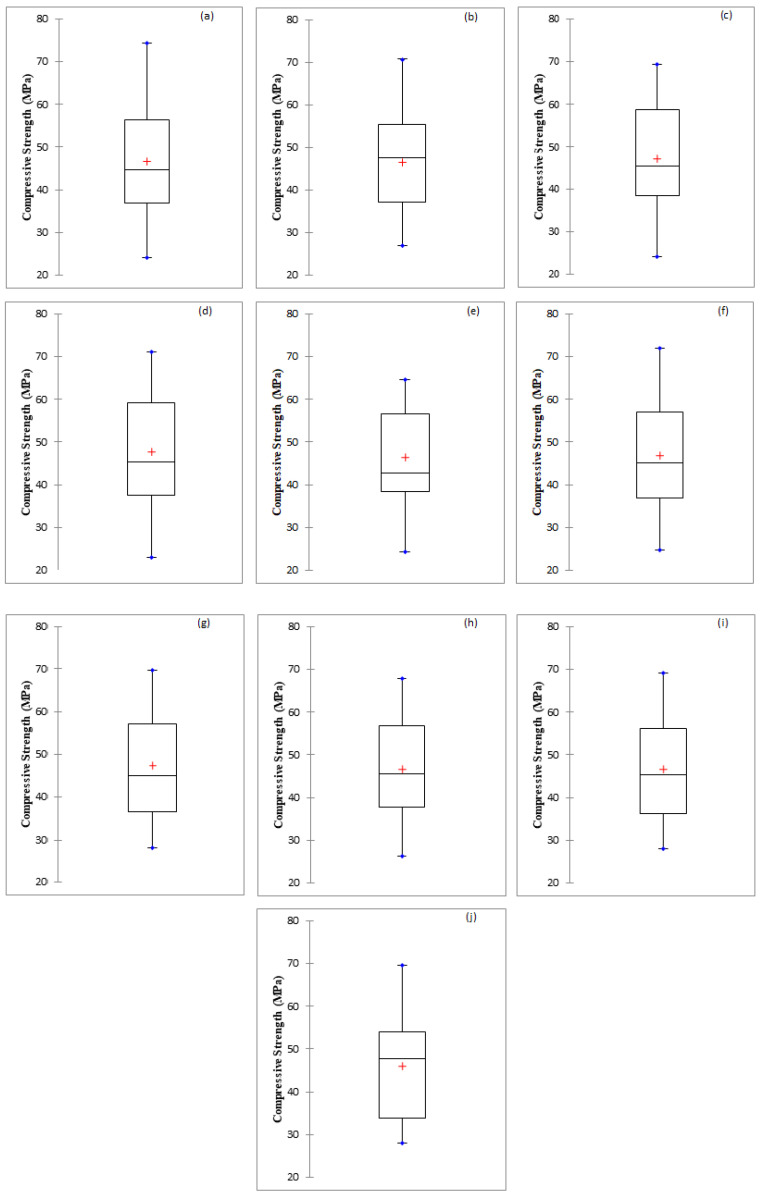
Box plot diagram (**a**) Actual, (**b**) M5P, (**c**) RF, (**d**) RT, (**e**) REP Tree, (**f**) SVR_Poly, (**g**) SVR_NPoly, (**h**) SVR_PUK, (**i**) SVR_RBF and (**j**) MLR.

In this investigation predictive accuracy of M5P, RF, RT, REP, and SVR based models have been assessed for the prediction of the compressive strength of bacterial concrete. The results have been compared with the multiple linear regression-based model (Table 6). For this purpose, seven statistical performance indices are selected namely CC, R^2^, RMSE, MAE, Bias, SI, and NSE. The results of performance evaluation indices recommend that polynomial kernel function-based support vector regression-based model performed better than other soft computing and regression-based models with CC (0.9919, 0.9901), R^2^ (0.9839, 0.9803), NSE (0.9832, 0.9800), RMSE (1.5680, 1.9384), MAE (0.7854, 1.5155), Bias (0.2353, 0.1350) and SI (0.0347, 0.0414) for training and testing stages, respectively. Overall SVR based models work better than a tree and MLR-based models except for SVR_NPoly based model. In tree-based models, RF based model is performing better than other tree based applied models. Overall MLR-based model performance is the least among all applied models with CC (0.9373), R^2^ (0.8786), RMSE (4.8727), MAE (3.9642), Bias (−0.7300), SI (0.1041), and NSE (0.8735) using testing data. Box plot diagram for all used techniques have been shown in Figure 4. Descriptive statistics results of actual and predictive values of compressive strength of concrete have been presented in Table 7.

**Figure 5:**
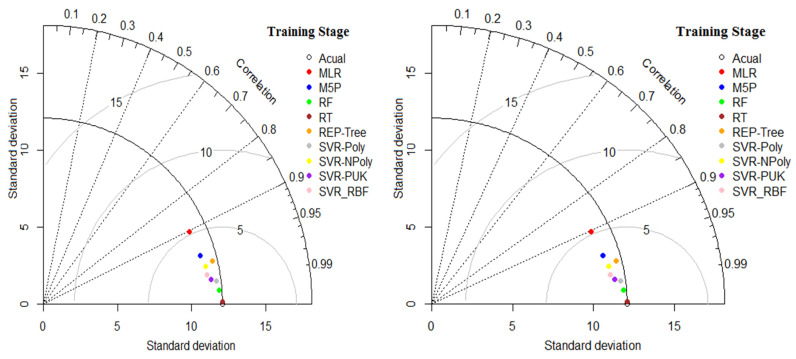
Taylor diagram for all applied models.

The Taylor chart shows the performance of the developed model in terms of correlation, RMSE and stand deviation, as shown in Figure 5. This figure indicates that SVR_Poly is the best performing model. Whereas, the MLR model has the worst performance, when using this data set to predict the compressive strength of concrete.

### 5.5. Sensitivity Analysis

The SVR_Poly model has been used for sensitivity testing to determine the important input variables in compressive strength. A set of different test data was created by deleting one input parameter at a time, as shown in Table 8. According to CC, MAE, and RMSE report, the impact of each input variable on the compressive strength was observed. The results in Table 8 show that, compared with other input parameters using this data set, curing time plays an important role in predicting the compressive strength of concrete.

## 6. Conclusions

In this investigation, Random Forest (RF), M5P, REP tree Random Tree and support vector regression have been developed to predict the compressive strength of concrete and compared with Multiple Linear Regression (MLR). For that purpose, the experiments have been performed with the variation of percentage of bacterial concrete 0%, 5%, 10%, and 15%, and two kinds of sand: natural sand and crushed aggregate.

The comparison analysis using performance evaluation indices concludes that developed RF approach outperformed rest of the tree based models and MLR (M5P, RT, REP tree and MLR) using given data set with CC (0.9973, 0.9868), R^2^ (0.9947, 0.9739), NSE (0.9944, 0.9722) and lower values of RMSE (0.9010, 2.2853), MAE (0.7387, 1.8161), Bias (0.0890, 0.2327) and SI (0.0199, 0.0488) respective for model development and validation period, correspondingly. Other major outcome from this investigation is the fact that RT based model performs better than M5P, REP Tree and MLR based models.

The comparison analysis using performance evaluation indices concludes that developed SVR_Poly approach outperformed rest of the models (SVR_Poly, SVR_NPoly, SVR_PUK, SVR_RBF and MLR) using given data set with CC (0.9919, 0.9901), R^2^ (0.9839, 0.9803) NSE (0.9832, 9800) and lower values of RMSE (1.5680, 1.9384), MAE (0.7854, 1.5155), Bias (0.2353, 0.1350) and SI (0.0347, 0.0414) for training and testing stages, respectively for model development and validation period, correspondingly. Other major outcome from this investigation is the fact that SVR_Poly based model performs better than SVR_NPoly, SVR_PUK, SVR_RBF, and MLR model. The results of the sensitivity study conclude that the curing time plays an important role in predicting the compressive strength of bacterial concrete using this data set with SVR_Poly model.

## Figures and Tables

**Table 1:** Physical properties of Portland cement.

S. No.	Test Property	Result	Requirements as per IS 12269-1987
1	Fineness (a) Sieve test (b) Blaine		
		2%	Not more than 10%
		285 m^2^/kg	Min 225 m^2^/kg
2	Normal Consistency	31.0%	-
3	Specific Gravity	3.01	-
4	Initial setting time	95 min	Not less than 30 min
5	Final setting time	284 min	Not more than 600 min
6	Compressive strength (a) 3 days (b) 7 days (c) 28 days		
		28 N/mm^2^	27 N/mm^2^ (min)
		41 N/mm^2^	37 N/mm^2^ (min)
		56 N/mm^2^	53 N/mm^2^ (min)
7	Soundness(Le-Chatlier Exp.)	2 mm	Not more than 10 mm

**Table 2:** Properties of coarse aggregate.

S. No.	Property	Test Value
1	Specific Gravity	2.71
2	Water absorption	0.5%
3	Sieve Analysis Test results	Grading Curve shown in Graph 3.2
4	Aggregate Impact Value, %	21.50
5	Aggregate crushing value, %	20.40
6	Combined Flakiness & Elongation Value, %	21.90

**Table 3:** The proportion of ingredients per one cubic meter of M20 grade concrete.

Mixture No	RBC00	RBC05	RBC10	RBC15	CBC00	CBC05	CBC10	CBC15
Cement (kg/m^3^)	340	340	340	340	340	340	340	340
River Sand (kg/m^3^)	736	736	736	736	-	-	-	-
Crushed Rock Sand (kg/m^3^)	-	-	-	-	736	736	736	736
Coarse Aggregate (kg/m^3^)	1214	1214	1214	1214	1214	1214	1214	1214
w/c ratio	0.48	0.48	0.48	0.48	0.48	0.48	0.48	0.48
Bacterial Cells (CFU/mL)	10^5^	10^5^	10^5^	10^5^	10^5^	10^5^	10^5^	10^5^
Percent of bacterial solution	00	05	10	15	00	05	10	15

**Table 4:** Proportion of ingredients per one cubic meter of M40 grade concrete.

Mixture No	RBC00	RBC05	RBC10	RBC15	CBC00	CBC05	CBC10	CBC15
Cement (kg/m^3^)	390	390	390	390	390	390	390	390
River Sand (kg/m^3^)	642	642	642	642	-	-	-	-
Crushed Rock Sand (kg/m^3^)	-	-	-	-	642	642	642	642
Coarse Aggregate (kg/m^3^)	1261	1261	1261	1261	1261	1261	1261	1261
w/c ratio	0.42	0.42	0.42	0.42	0.42	0.42	0.42	0.42
Bacterial Cells (CFU/mL)	10^5^	10^5^	10^5^	10^5^	10^5^	10^5^	10^5^	10^5^
Percent of bacterial solution	00	05	10	15	00	05	10	15

RBC: Bacterial concrete with river sand. CBC: Bacterial concrete with crushed stone sand.

**Table 5:** Features of data set used the model development and validation.

Input and Output Parameters	Mean	Standard Deviation	Minimum	Maximum	Confidence Level (95.0%)	Data Set
Cement	365.00	25.10	340	390	4.39	Overall
	364.71	25.14	340	390	5.36	Training
	365.61	25.30	340	390	7.99	Testing
Sand	689.00	47.18	642	736	8.25	Overall
	689.54	47.27	642	736	10.07	Training
	687.85	47.57	642	736	15.01	Testing
Aggregate	1237.50	23.59	1214	1261	4.12	Overall
	1237.23	23.63	1214	1261	5.03	Training
	1238.07	23.78	1214	1261	7.50	Testing
W/C	0.45	0.03	0.42	0.48	0.01	Overall
	0.45	0.03	0.42	0.48	0.01	Training
	0.45	0.03	0.42	0.48	0.01	Testing
Curing period	126.75	124.35	7	365	21.75	Overall
	124.97	124.29	7	365	26.49	Training
	130.54	125.93	7	365	39.75	Testing
BC	0.08	0.06	0	0.15	0.01	Overall
	0.07	0.06	0	0.15	0.01	Training
	0.08	0.06	0	0.15	0.02	Testing
Kind of Sand	1.50	0.50	1	2	0.09	Overall
	1.47	0.50	1	2	0.11	Training
	1.56	0.50	1	2	0.16	Testing
Compressive strength (MPa)	45.70	12.70	21.56	74.46	2.22	Overall
	45.17	12.16	21.56	71.12	2.59	Training
	46.82	13.87	24.16	74.46	4.38	Testing

**Table 6:** Performance evaluation parameters M5P, RF, RT, REP Tree, SVR_Poly (Polynomial kernel), SVR_NPoly (Normalized Poly Kernel), SVR_PUK, SVR_RBF (RBF Kernel), MLR.

Approaches	CC	R^2^	RMSE	MAE	Bias	SI	NSE
	Training Stage						
M5P	0.96	0.92	4.90	2.77	0.20	0.11	0.92
RF	1.00	0.99	0.90	0.74	0.09	0.02	0.99
RT	1.00	1.00	0.13	0.06	0.00	0.00	1.00
REP Tree	0.97	0.94	2.89	2.32	0.00	0.06	0.94
SVR_Poly	0.99	0.98	1.57	0.79	0.24	0.03	0.98
SVR_NPoly	0.98	0.95	2.78	1.67	0.65	0.06	0.95
SVR_PUK	0.99	0.98	1.90	0.80	0.66	0.04	0.98
SVR_RBF	0.99	0.97	2.27	1.06	0.69	0.05	0.96
MLR	0.90	0.82	5.19	4.22	0.00	0.11	0.82
	**Testing Stage**						
M5P	0.97	0.94	4.88	2.88	−0.10	0.10	0.93
RF	0.99	0.97	2.29	1.81	0.23	0.05	0.97
RT	0.98	0.96	2.82	2.49	0.86	0.06	0.96
REP Tree	0.96	0.92	3.81	2.97	−0.35	0.08	0.92
SVR_Poly	0.99	0.98	1.94	1.52	0.14	0.04	0.98
SVR_NPoly	0.98	0.96	2.96	2.36	0.63	0.06	0.95
SVR_PUK	0.99	0.98	2.69	2.06	−0.19	0.06	0.96
SVR_RBF	0.98	0.97	3.30	2.59	−0.27	0.07	0.94
MLR	0.94	0.88	4.87	3.96	−0.73	0.10	0.87

**Table 7:** Descriptive statistics results of actual and predictive values of compressive strength of concrete [MPa].

Statistic	Actual	MLR	M5P	RF	RT	REP Tree	SVR_Poly	SVR_NPoly	SVR_PUK	SVR_RBF
Minimum	24.16	27.95	27.08	23.99	23.02	24.33	24.78	28.15	26.27	28.00
Maximum	74.46	69.55	70.85	69.32	71.12	64.73	72.02	69.59	67.85	69.24
1st Quartile	36.98	33.75	37.25	38.36	37.64	38.43	36.95	36.71	37.86	36.29
Median	44.76	47.82	47.75	45.36	45.43	42.66	45.20	45.07	45.46	45.37
3rd Quartile	56.37	54.01	55.46	58.70	59.12	56.61	56.98	57.13	56.76	56.25
Mean	46.82	46.09	46.72	47.05	47.68	46.48	46.96	47.45	46.63	46.55
IQR	19.39	20.26	18.20	20.34	21.48	18.18	20.04	20.42	18.90	19.96

**Table 8:** Sensitivity results using SVR_Poly based model.

Input Variable Combination							Target Variable	SVR_Poly		
Cement	Sand	Aggregate	W/C	Curing	BC	Kind of Sand	Compressive Strength (MPa)	CC	MAE	RMSE
								0.99	1.52	1.94
								0.98	1.82	2.47
								0.98	2.04	2.70
								0.98	1.82	2.45
								0.98	2.04	2.70
								0.80	7.21	8.59
								0.96	3.28	3.89
								0.98	2.48	2.88

## Data Availability

The data presented in this article are available within the article.
